# Co Nanoparticle-Encapsulated Nitrogen-Doped Carbon Nanotubes as an Efficient and Robust Catalyst for Electro-Oxidation of Hydrazine

**DOI:** 10.3390/nano11112857

**Published:** 2021-10-26

**Authors:** Hui Wang, Qing Dong, Lu Lei, Shan Ji, Palanisamy Kannan, Palaniappan Subramanian, Amar Prasad Yadav

**Affiliations:** 1 College of Biological, Chemical Sciences and Engineering, Jiaxing University, Jiaxing 314001, China; wangh@qust.edu.cn (H.W.); jishan@mail.zjxu.edu.cn (S.J.); 2 College of Chemical Engineering, Qingdao University of Science and Technology, Qingdao 266042, China; dongqing125800@163.com; 3 New Technologies–Research Center, University of West Bohemia, 30100 Plzeň, Czech Republic; 4 Central Department of Chemistry, Tribhuvan University, Kirtipur 44613, Nepal; amar2y@yahoo.com

**Keywords:** Co nanoparticles, N-doped carbon nanotubes, hydrazine, electro-oxidation, fuel cell

## Abstract

Structural engineering is an effective methodology for the tailoring of the quantities of active sites in nanostructured materials for fuel cell applications. In the present study, Co nanoparticles were incorporated into the network of 3D nitrogen-doped carbon tubes (Co@NCNTs) that were obtained via the molten-salt synthetic approach at 800 °C. Morphological representation reveals that the Co@NCNTs are encompassed with Co nanoparticles on the surface of the mesoporous walls of the carbon nanotubes, which offers a significant active surface area for electrochemical reactions. The CoNPs/NCNTs-1 (treated with CaCl_2_) nanomaterial was used as a potential candidate for the electro-oxidation of hydrazine, which improved the response of hydrazine (~8.5 mA) in 1.0 M NaOH, as compared with CoNPs/NCNTs-2 (treated without CaCl_2_), NCNTs, and the unmodified GCE. Furthermore, the integration of Co helps to improve the conductivity and promote the lower onset electro-oxidation potential (−0.58 V) toward the hydrazine electro-oxidation reaction. In particular, the CoNPs/NCNTs-1 catalysts showed significant catalytic activity and stability performances i.e., the i-t curves showed notable stability when compared with their initial current responses, even after 10 days, which indicates the significant durability of the catalyst materials. This work could present a new approach for the design of efficient electrode materials, which can be used as a favorable candidate for the electro-oxidation of liquid fuels in fuel cell applications.

## 1. Introduction

Energy demands, climate transformation, and environmental effluence are the key factors to consider in the search for alternative fossil fuels with greener, more sustainable, and cleaner energy sources [1,2]. Hence, a collection of small organic compounds (e.g., ethanol, methanol, glycerol, and ethylene glycol) have been selected as potential liquid-fuel contenders in direct alcohol fuel cells [3,4,5,6,7]. Though, the incomplete oxidation of small organic molecules can be accumulated on intermediate products, thereby considerably diminishing fuel cell efficacy. Thus, direct hydrazine fuel cell (DHFC) technology was adopted for the use of a greener, carbon-less fuel known as hydrazine (N_2_H_4_), which has a higher hydrogen content (12.5 wt.%) than sodium borohydride (10.6 wt.%), and is comparable to methanol; hydrazine has received considerable recognition in fuel cell technology [8,9,10,11,12,13]. Furthermore, hydrazine shows significant benefits, including a larger theoretical cell-voltage (1.56 V vs. SHE), environmentally harmless final-products (only N_2_ and H_2_O), a higher energy density (5500 W h L^−1^), and easy transportation and storage [14,15,16]. Practical applications of DHFCs are mainly affected because of the slower kinetic and higher onset oxidation potential related to the direct electro-oxidation of hydrazine at unmodified electrodes [17,18]. Thus, noble-metal-based (Pt, Pd, Au, and Ag) transducers were extensively used as potential electro-catalysts in several typical hydrazine fuel cells; consequently, such fuel cells are expensive and challenging to fabricate for widespread commercial use [19,20,21,22,23,24,25]. Particularly, the Pt- or Pd-nanostructure-based electro-catalytic oxidation reaction of hydrazine displayed other cross-reactions, i.e., the oxidation of H_2_, as an alternative to the complete electro-oxidation of hydrazine [26,27,28].

The thermodynamic voltage that corresponds to the complete oxidation of hydrazine to nitrogen and water is E_0_ = 0.496 V vs. RHE at pH 14. A high voltage closer to the thermodynamic cell voltage of E_0_ = 1.56 V can be attained in a direct hydrazine fuel cell only when the electrocatalytic anode material is able to oxidize hydrazine at a low overpotential. Therefore, it is vital to fabricate highly active, viable, and low-cost anode catalysts with a low overpotential for the demonstration of direct hydrazine fuel cells for different applications. In recent times, supported non-noble metal catalysts have been effectively prepared, and have progressively replaced the customary fuel cell catalysts that contain noble (precious) metals and their corresponding metal alloys in the electro-oxidation reaction of hydrazine [29,30,31,32,33,34,35,36]. For instance, Duan and co-workers established a novel type of bendable electrode that was constructed using Cu nanocubes that were inserted into graphene paper via a simple electro-deposition process. The Cu/GP composite showed a significant electro-catalytic response with an onset oxidation potential of −0.10 V (0.936 V vs. RHE; overpotential is 0.44 V) towards the electro-oxidation of hydrazine, which acted as a catalyst for DHFCs [35]. Subsequently, Liu et al. fabricated a graphene/Cu electro-catalyst through a simple cyclic voltammetric electrolysis method. The obtained 3D-sandwich type of RGO/Cu nanocomposite (ST-RGO/Cu), which was composed of Cu nanoparticles on the surface of RGO sheets, showed an enhanced electro-chemically accessible surface area for the improved (in terms of current density and low onset overpotential) electro-oxidation of hydrazine in an alkaline solution [33]. Later, Khilari et al. reported a one-step hydrothermal preparation method to integrate MnFe_2_O_4_ nanoparticles with an N-doped N-rGO (MnFe_2_O_4_ NPs/N-rGO) as a bi-functional electro-catalyst for both the anodic electro-oxidation of hydrazine with an onset oxidation potential of −0.20 V (0.836 V vs. RHE; overpotential is 0.34 V), and the cathodic reduction reaction of oxygen [34]. Recently, Wang et al. reported the sequence preparation of a carbon-material-based, phosphatized Cu-Ni (P-Cu_x_Ni_y_/C) for the electro-oxidation of hydrazine through the high-temperature phosphating route. Considering this series of catalysts, the P-Cu_2_Ni/C catalyst showed superior activity towards the hydrazine oxidation reaction with an onset potential of −0.54 V (0.496 vs. RHE; overpotential is 0 V) [32]. Inspired by the above synthetic methods, in this work, we report a molten-salt-method-based synthetic approach to obtain Co nanoparticles that are inserted within the surface walls of an N-doped carbon nanotube network (Co@NCNTs). Our findings show that the simple and efficient synthetic method was established without the use of corrosive or structure-directing agents. Notably, CaCl_2_ and melamine were employed as a molten-salt medium, and a nitrogen and carbon source, respectively. Under optimized experimental conditions, the Co@NCNTs-1 (with CaCl_2_ salt) catalyst shows a tremendous electro-catalytic response and robustness toward the electro-oxidation of hydrazine in 1.0 M NaOH. In addition, the catalytic activity of the Co@NCNTs-1 sample is highly comparable with those of the Co@NCNTs-2 (without CaCl_2_ salt) and NCNTs, and other nanocomposite materials.

## 2. Experimental Part

### 2.1. Reagents

Cobalt chloride hexahydrate (CoCl_2_.6H_2_O), melamine, calcium chloride (CaCl_2_), nitric acid (HNO_3_), hydrazine (N_2_H_4_), sodium hydroxide (NaOH), sulfuric acid (H_2_SO_4_), urea, and ethanol (EtOH) were purchased from Aladdin Chemicals Ltd., Shanghai, China.

### 2.2. Synthesis of Co@NCNTs, and NCNTs Nanomaterials

The Co nanoparticle-inserted NCNTs were prepared as follows [37]; 1.5 g of CoCl_2_.6H_2_O and 2.0 g of melamine were mixed in 50 mL of absolute ethanol, and ultrasonically processed for 30 min. Then, the above reaction precursors were treated at 45 °C for 1 h to eliminate the ethanol solvent. Next, 0.28 g of CaCl_2_ salt was added into the above sample and ball-milled at a rate of 550 rpm for 6 h in a zirconia vessel with zirconia balls. The ball-milled reactants were placed in a porcelain container, treated at 800 °C under a steady flow of inert N_2_ for 3 h, and then cooled down to room temperature. The obtained sample was processed in 1.0 M HNO_3_ for 24 h to eliminate any unstable Co species. Finally, the above sample was rinsed with Millipore water until a neutral pH value was obtained, then it was desiccated at 60 °C for 12 h. Elsewhere, in order to study the influence of the molten Ca salt on the final nanostructure of the Co@NCNTs, the catalyst sample was also synthesized without the addition of CaCl_2_ into the early reaction mixture. The obtained catalyst samples were labelled as Co@NCNTs-1 (with CaCl_2_) and Co@NCNT-2 (without CaCl_2_), respectively. For comparison analysis, standard carbon nanotubes (diameter: 10–30 nm, length: 2 mm, actual surface area: 160–200 m^2^g^−1^), were received from Namigang Corp. Shenzhen, China. Standard CNTs (1.0 g) were treated with 5.0 mL of HNO_3_ and 30 mL of H_2_SO_4_, and refluxed at 80 °C for 2 h. Finally, the obtained CNT samples were collected and washed with Milli-Q water. Subsequently, 50 mg of pre-treated CNTs were mixed into 5 g of urea and heated to 600 °C to obtain the NCNTs. Urea begins to decay at 160 °C, and the entire decaying process is completed at 600 °C, while melamine begins to decompose at 350 °C and, notably, is not totally decomposed at 600 °C. Thus, urea can be used as a nitrogen resource in this study. The collected sample was labelled as NCNTs.

### 2.3. Characterization of Nanomaterials

The sizes and surface morphologies of the as-prepared nanomaterials were characterized by Carl Zeiss Ultra Plus FE-SEM (Oberkochen, Germany), and a JEOL JEM-2000 FX TEM instrument (Applied Rigaku Technologies, Inc., Austin, TX, USA) working at 200 kV. The specific surface area and corresponding pore-size distribution were examined by using a Quantachrome Autosorb-IQ volumetric analyzer (Anton Paar Trading Co. Ltd, Shanghai, China). Raman spectra were measured on an RFS 100 Ft-Raman spectroscope (Bruker, Riverview, FL, USA) at a 532.0 nm excitation of the laser beam. The XRD technique was used to characterize the crystalline nature of the as-prepared samples using a Shimadzu XDe3A spectrometer (Malvern Panalytical B.V., Almelo, The Netherlands) with a source of Ni-Cu-Kα (λ = 0.15418 nm) that was applied at 40 kV and 30 mA. X-ray photoelectron spectra (XPS) were acquired in order to study the chemical composition of the nanomaterials using VG Escalab 210 spectrometer (Malvern Panalytical B.V., Almelo, The Netherlands) equipped with an Mg 300 W X-ray. Inductively coupled plasma-optical emission spectroscopy (ICP-OES) measurements were performed on a Varian Vista MPX instrument (SpectraLab Scientific Incorporation, Alexandria, VA, USA).

### 2.4. Electrochemical Measurements

The electro-oxidation of hydrazine was carried out on a CHI660D from CH Instruments (CHI 660, CH Instruments, Inc., Shanghai, China). A glassy-carbon electrode (GCE; 3 mm diameter) was used as the working electrode, which was prepared as follows; 5.0 mg of Co@NCNTs-1 catalyst material was uniformly dissolved into 1 mL ethanolic-nafion (0.25 wt%) by ultrasound mixing for 30 min. Then, 8 μL of the above catalyst was placed onto the surface of the GCE with ~0.204 mg cm^−2^ catalyst loading and dried out under argon atmosphere. For comparison purposes, the Co@NCNTs-2 and NCNTs catalysts were used for experimental studies. Ag/AgCl (KCl), and Pt wire were used as a reference and counter electrode, respectively. A solution of 1.0 M NaOH purged with argon gas was used as a supporting electrolyte. EIS was carried out using all of the catalyst electrodes in 1.0 M NaOH and 0.1 M hydrazine with a window of 1.0 Hz to 1.0 MHz and an amplitude of 5 mV.

## 3. Results and Discussion

### 3.1. Characterization of Co@NCNTs Nanomaterials

**Figure 1:**
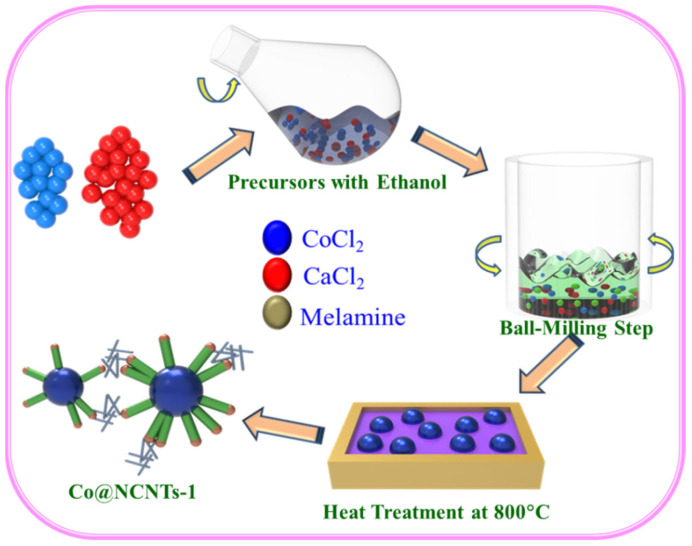
Representation showing the synthetic steps of Co@NCNTs−1 catalyst sample.

**Figure 2:**
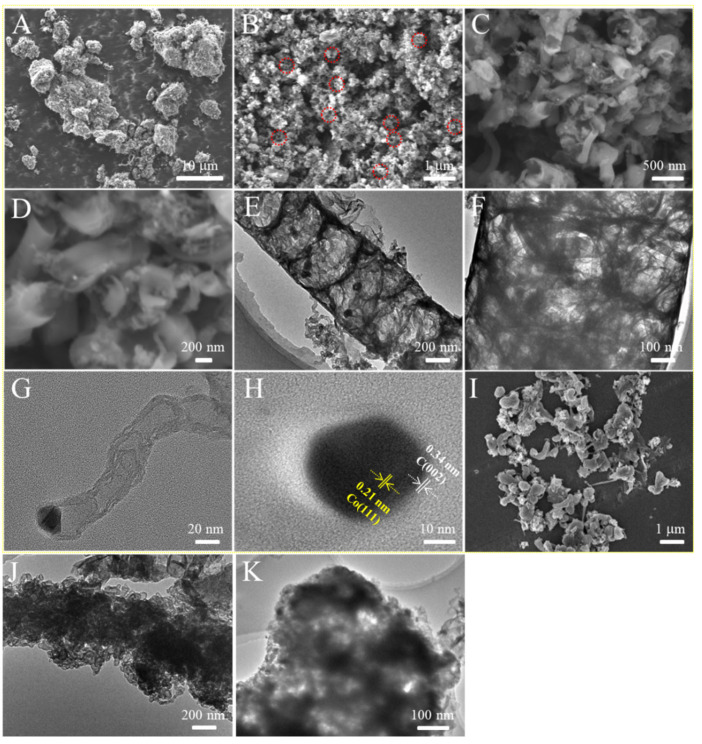
SEM (**A**–**D**), and corresponding TEM images (**E**–**H**) of Co@NCNTs−1 catalyst sample prepared in the presence of CaCl_2_. SEM (**I**), and TEM (**J**,**K**) images showing the Co@NCNTs−2 catalyst sample prepared in the absence of CaCl_2_.

The synthetic procedure for Co@NCNTs samples is diagrammatically shown in Figure 1, representing the easiness of the single-step pyrolysis process that was employed for the precursors. The commercially available, low-cost melamine compound was chosen as the carbon and nitrogen source in order to obtain N-doped carbon material, and CaCl_2_ was used as a molten-salt model for the carbonization of melamine. Next, the precursors were heated to 800 °C, at which point the CaCl_2_ melted and produced nano-droplets, and then dispersed into the carbon precursor sample during pyrolysis, which resulted in the formation of pores on the surfaces of the carbonized materials. Consequently, CaCl_2_ was eliminated by the rinsing process, which was considerably easier compared to some of the more complex steps involved in the traditional template-based methods [38,39]. This single step, low-cost synthetic method for obtaining Co@NCNT samples in a large-scale environment that can then be used as potential electro-catalysts in many fuel cell applications. The surface morphology and microstructure of Co@NCNTs-1 were characterized by SEM and TEM image analyses as displayed in Figure 2. Low-resolution SEM images of Figure 2A,B displayed many isolated, irregular microstructures with rough surfaces.

Notably, high-resolution SEM images (Figure 2C,D) showed tube-like nanostructures with irregular Co particles that were interconnected on the surface walls of the NCNTs. It is important to mention here that the embedded Co nanoparticles were hard to visualize using SEM characterization. Thus, we carried out TEM image analysis, as shown in Figure 2E–H, which clearly revealed the formation of Co nanoparticles incorporated on the surface of the NCNTs. Furthermore, many mesopores were seen in the hollow NCNT nanostructure, which exposed large active sites and improved electron-transfer kinetics (Figure 2E,F). The HR-TEM images displayed that the NCNTs were smooth, tube-like nanostructures that were encompassed by spherically shaped Co nanoparticles (25 ± 2 nm) within the surface walls of the hollow NCNT structure (Figure 2G,H). In addition, lattice fringes with a *d*-spacing of 0.21 nm were ascribed to the Co(111) lattice, and demonstrated the existence of the spherically shaped Co nanoparticles (Figure 2H). The SEM image of the NCNTs alone showed tubular smooth walls and no Co nanoparticles on their surfaces (Figure S1). Furthermore, the SEM (Figure 2I), and TEM (Figure 2J,K) images displayed many irregularly aggregated nanostructures with rough surfaces formed without the addition of CaCl_2_ in the precursor mixture, which indicates the critical role of CaCl_2_ during the formation of tube-like NCNTs.

**Figure 3:**
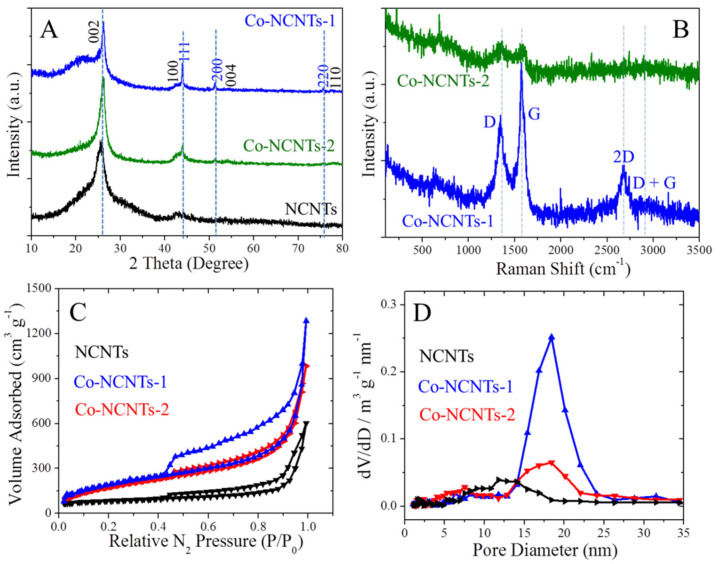
(**A**) XRD patterns obtained for NCNTs, Co@NCNTs−1, and Co@NCNTs−2 catalysts samples. (**B**) Raman spectral data obtained for Co@NCNTs−1, and Co@NCNTs−2 catalysts samples. (**C**) N_2_ adsorption-desorption isotherms, and corresponding pore size distributions (**D**) of NCNTs, Co@NCNTs−1 and Co@NCNTs−2 catalysts samples.

**Figure 4:**
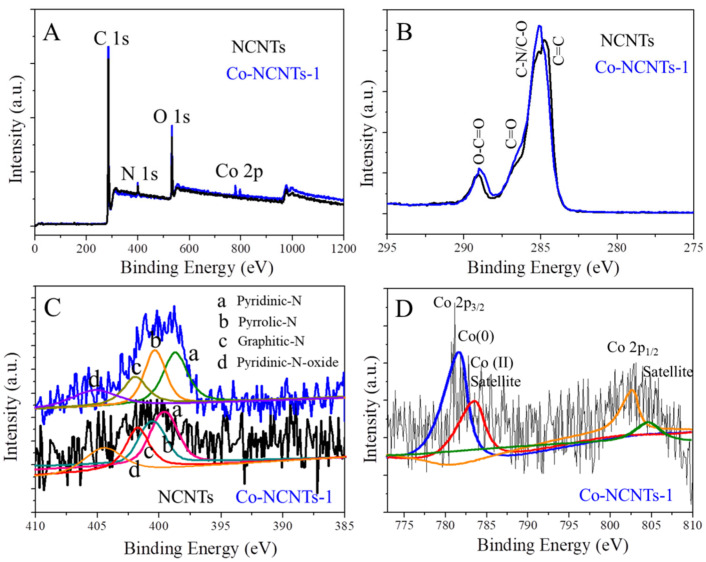
(**A**) XPS survey spectra of NCNTs and Co@NCNTs−1; (**B**) high resolution XPS spectra of C 1s (**B**), N 1s (**C**), C (**D**) and Co 2p elements.

Next, the crystalline nature of the Co@NCNTs-1 and Co@NCNTs-2 catalyst samples were examined by XRD analysis as displayed in Figure 3A. An intense diffraction peak that was observed at ca. 26.2° was mainly attributed to the (002) plane of the carbon substrate [38] from the NCNTs, which indicates the presence of graphitic carbon. Other diffraction peaks at ca. 44.3°, 51.6° and 76.2° were ascribed to the (111), (200) and (220) planes of the face-centered-cubic (*fcc*) Co^0^ phase (JCPDF#15-0806) in the Co nanoparticles, which evidently supported the TEM results. Particularly, the Co@NCNTs-2 catalyst sample did not show a notable XRD pattern when compared with the Co@NCNTs-1 catalyst sample, which demonstrates the distinct nanocrystalline nature of the sample. The Co@NCNTs-1 and Co@NCNTs-2 catalyst samples were further analyzed by the Raman spectroscopy method. The corresponding results were shown in Figure 3B, where all the Raman spectra commonly displayed D- and G-bands located at ca. 1340 cm^−1^ at ca. 1580 cm^−1^, respectively. D- and G-bands were mainly the result of a disordered structure, and the sp^2^-hybridized graphitic carbon component of the Co@NCNTs-1 catalyst sample, respectively [39]. Hence, the intensity ratios of D- (*I*_D_) and G-bands (*I*_G_) were used to assess the surface defects in the catalyst samples. It has been reported that the higher value of *I*_D_/*I*_G_ resulted in high-density structural defects on the surface. The Co@NCNTs-1 (0.98) catalyst sample showed a higher *I*_D_/*I*_G_ ratio than the Co@NCNTs-2 sample (0.74), which revealed the existence of prior high-density surface defects in the nanomaterial, which offered more active sites for electro-oxidation reactions, thereby ensuing an enhanced electro-catalytic response. In addition, the 2D peak at ca. 2795 cm^−1^ was seen in the spectrum of Co@NCNTs-1, indicating the higher graphitization of the sample [40]. The specific surface areas of all nanomaterials were derived from the BET method (Figure 3C). The Co@NCNTs-1 showed a higher specific surface area, 282.1 m^2^g^−1^, than the other samples (187.8 m^2^g^−1^ for Co@NCNTs-1 and 171.3 m^2^g^−1^ for NCNTs). The obtained BET surface area was comparable with the recently reported N-doped CNT materials (Table S1). The porosity of the NCNTs, Co@NCNTs-1 and Co@NCNTs-2 was assessed by N_2_ adsorption-desorption curves, which are displayed in Figure 3C. Based on the IUPAC nomenclature, the N_2_ adsorption-desorption curves of all three nanomaterials were of the hybrid I/IV-type with a hysteresis loop at the high pressure region, which indicates the existence of both micropores and mesopores [41]. Notably, a large hysteresis loop was observed for the Co@NCNTs-1 sample (blue line), when compared with the Co@NCNTs-2 (red line) and the NCNT (black line) samples, owing to its highly mesoporous structure. As can be seen in Figure 3D, the pore size was dispersed over a wide range for NCNTs and the Co@NCNTs-2; but, a narrow range was observed for the Co@NCNTs-1, i.e., pore sizes were positioned at ca. 18 nm, indicating that the presence of molten CaCl_2_ had efficiently supported the development of mesopores during the carbonization process. Usually, several micropores in a nanomaterials are not easily reachable by reactants, therefore the outside surface area was calculated through a V-t plot [41] (247.2 m^2^g^−1^ for Co@NCNTs-1, 179.4 m^2^g^−1^ for Co@NCNTs-1, and 156.1 m^2^g^−1^ for NCNTs). Thus, the defined variables were adopted in order to characterize the actual surface area that would be accessible for electro-oxidation reactions (Figure 3C,D); particularly, Co@NCNTs-1 showed a higher external surface area, which displayed larger active sites, and subsequently improved the electro-catalytic activity in contrast to Co@NCNTs-2 and NCNTs. The existence of relatively low external surface area in later samples was possibly due to the absence of Co nanoparticles in Co@NCNTs-2. In addition, the Co loading of the Co@NCNTs-1 and Co@NCNTs-2 catalyst samples were performed using inductively coupled plasma-optical emission spectroscopy (ICP-OES). The Co loading values (Co wt%) were 7.3 and 3.4 for Co@NCNTs-1 and Co@NCNTs-2 catalyst samples, respectively. The chemical state and composition of the as-prepared catalyst nanomaterials were characterized by XPS analysis. Figure 4A clearly shows the survey XPS of NCNTs and that the Co@N-CNTs-1 catalyst materials are generally composed of N, O, and C elements. In addition, notable Co signals observed at 781 eV and 802 eV in the XPS spectrum of Co@NCNsT-1 (Figure 4A, blue line) indicated that the Co nanoparticles were wrapped on the surface walls of the NCNT materials.

Next, the XPS of C 1s in NCNTs (Figure 4B; black line), and Co@NCNTs-1 (Figure 4B; blue line) showed four peaks at 284.6, 285.3, 286.6 and 289.1 eV, which were related to C=C, C-O/C-N, C=O and O-C=O groups, respectively [41]. The existence of a C-N signal proved that the nitrogen element had inserted into the tubular carbon structures and bonded with carbon. Thus, the majority of carbon is present as the C-O/C-N form in the carbon nanotube; in particular, nitrogen species play a vital part in the improvement of the electro-activity of N-doped carbon nanomaterials. XPS spectra relating to N 1s were fitted into four peaks at 399.6, 401.4, 403.6, 404.2 and 406.8 eV corresponding to pyridinic-N, pyrrolic-N, graphitic-N, and pyridinic N-oxide, respectively (Figure 4C) [41]. Notably, the 2p peaks of Co(Co 2p_3/2_ and Co 2p_5/2_), and its corresponding shake-up satellite peaks observed at ~781.0 eV and 802.0 eV, revealed that the Co@NCNTs-1 catalyst was embedded with Co nanoparticles as active species in the NCNT walls (Figure 4D). Pyrrolic-N and pyridinic-N are usually known as the active centers of N-doped carbons [42], which offer electrons during the electro-oxidation processes [43]; thus, large numbers of these species typically showed higher electro-catalytic responses. The actual content of pyrrolic-N and pyridinic-N in the Co@NCNTs-1 sample (33.8%) was larger than in the NCNTs, demonstrating the possible higher electro-catalytic activity of Co@NCNTs-1.

### 3.2. Electro-Oxidation of Hydrazine at NCNTs and Co@NCNTs

**Figure 5:**
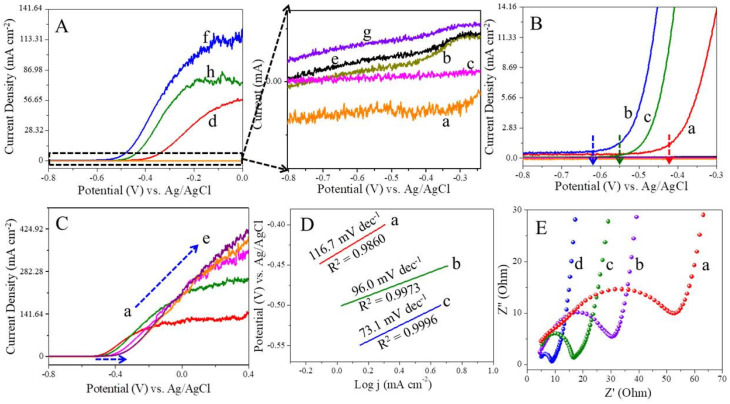
(**A**) LSV polarization curves obtained for bare GCE in the absence (curve a), and presence of 0.10 M hydrazine (curve b); The GCE modified with NCNTs, Co@NCNTs−1, and Co@NCNTs−2 catalysts in the absence (curves c, e, and g), and the presence of 0.10 M hydrazine (curves d, f, and h) in 1.0 M NaOH solution at a scan rate of 10 mV s^−1^. (**B**) NCNTs (curve a), Co@NCNTs−1 (curve b), and Co@NCNTs−2 (curve c) modified GCEs showing exact “onset” polarization curves of hydrazine electro-oxidation. (**C**) LSV polarization curves obtained at Co@NCNTs−1 electrode in 1.0 M (a), 2.0 M (b), 3.0 (c), 4.0 (d), and 5.0 M (e) NaOH solution containing 0.1 M hydrazine at a scan rate of 10 mV s^−1^. (**D**) Tafel plots taken on a NCNTs (curve a), Co@NCNTs−2 (curve b), and Co@NCNTs−1 (curve c) catalyst samples modified GCEs resulting from their respective LSV curves. (**E**) EIS responses obtained at bare GCE (curve a), NCNTs (curve b), Co@NCNTs−2 (curve c), and Co@NCNTs−1 (curve d) catalyst-modified GCEs in 1.0 M NaOH + 0.10 M hydrazine solution.

**Scheme 1:**
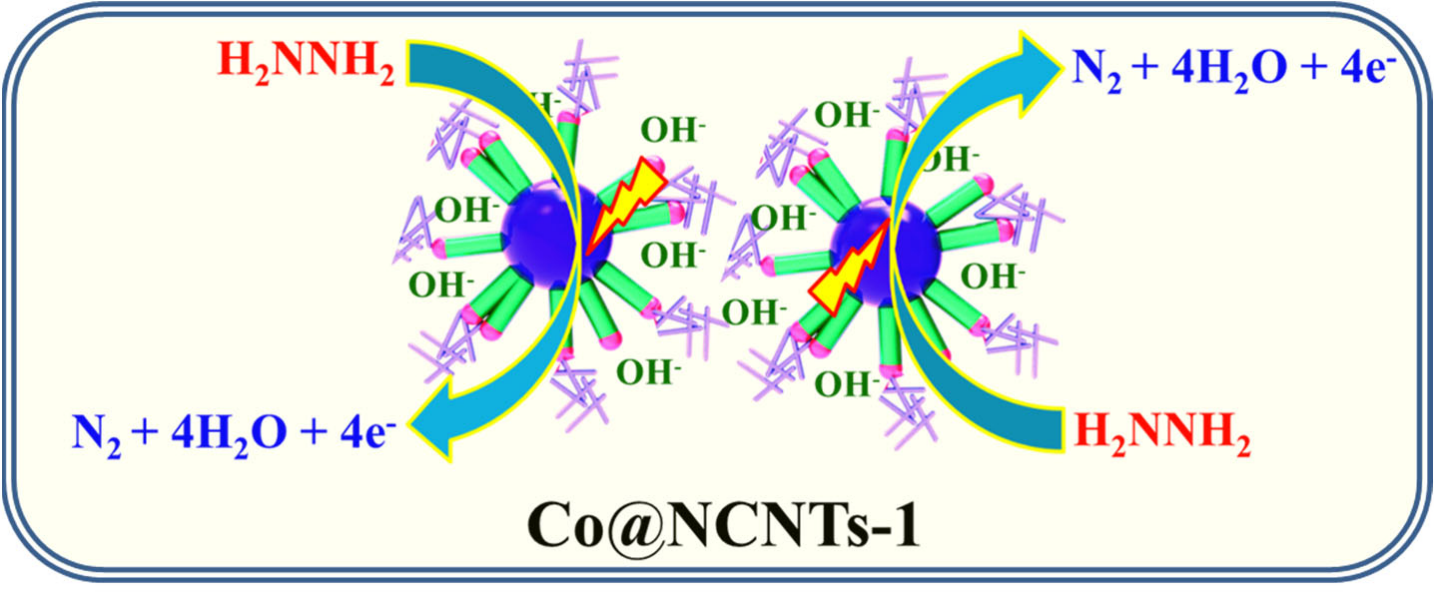
Graphical illustration displaying probable electro-oxidation mechanism of hydrazine at Co@NCNTs−1 surface.

The electro-oxidation responses of the as-prepared NCNTs and Co@NCNTs catalyst materials were examined by the LSV method in 1.0 M NaOH and 0.1 M hydrazine at a scan rate of 10 mV s^−1^ (Figure 5A). The LSV-polarization curves that were acquired for the bare GCE in the absence (curve a), and presence (curve b) of hydrazine, failed to display a significant electro-oxidation reaction of hydrazine due to absence of active sites on its surface. Next, the NCNT-catalyst-modified GCEs in the absence (curve c) of hydrazine displayed an identical response to the bare GCE; however, it displayed the noticeable electro-oxidation of hydrazine (~3.8 mA; Figure 5A: curve d) at −0.1 V with an onset oxidation at −0.42 V (see red color arrow mark in Figure 5B; curve a), indicating that the NCNTs possessed a small amount of electro-activity owing to their available active surface areas. Interestingly, the Co@NCNTs-1-catalyst-(with CaCl_2_)-modified GCE displayed no noticeable activity in the absence of hydrazine (Figure 5A; curve e) and a significant LSV polarization electro-oxidation response (~8.1 mA) toward the electro-oxidation of hydrazine (Figure 5A: curve f) at −0.2 V with an onset oxidation potential of −0.58 V (see blue color arrow mark in Figure 5B; curve b), which indicates the existence of Co nanoparticles in their components. The enhanced electro-oxidation of Co@NCNTs-1 was associated with: (i) the noticeable electronic conductivity ensuing from the Co nanoparticle-surrounded-NCNT carbon nanotubes that could efficiently enable the transportation of ions via its mesopores, which apparently used available active sites to attain a higher electro-oxidation of hydrazine; (ii) the fact that many Co nanoparticles were arbitrarily dispersed without any accumulation on the surface walls of Co@NCNTs-1, which has large active sites and can access numerous hydrazine molecules for an enhanced electro-oxidation reaction; (iii) the existence of a wall-like tubular surface morphology of the Co@NCNTs-1 catalyst sample with a higher content of pyridinic N and pyrrolic N (52.4%), which is higher than that of the NCNTs (34.1%), that could serve as an efficient catalytic center for the incorporation of many Co nanoparticles, thereby enhancing both the interior and exterior surface areas of the catalyst; (iv) the N-doped carbon-nanotube network that acts as an electron tank for the prompt electron transfer from the hydrazine molecule towards the surface of the catalyst. (v) the synergistic effect between Co nanoparticles and the surface walls of NCNTs that could have resulted in an enhanced electro-oxidation response. The Co^2+^ sites on the Co@NCNTs-1 catalyst principally initiate the adsorption of OH^−^ ions due to the robust electrostatic attraction (the availability of more unfilled d-orbitals in Co^2+^ species than in metallic Co), and catalyze the electro-oxidation as revealed in Scheme 1 [10,44]. Comprehensively, the adsorption of OH^−^ and hydrazine (N_2_H_4_) on the surface of Co@NCNTs-1 catalyst can be considered as the rate-determining step (N_2_H_4ads_ + OH^−^_ads_/N_2_H_3ads_ + H_2_O + e^−^) [45], and the adsorption of OH^−^ ions on the surface of Co sites might be the principal step for the electro-oxidation reaction of hydrazine. The Co@NCNTs-1 catalyst displays large available Co sites, as revealed by the Co 2p XPS spectra, which improves adsorption of the OH^−^ ions and facilitates a superior polarization reaction with a lower onset potential for electro-oxidation reaction of hydrazine [9,10].

The Co@NCNTs-2 catalyst (without CaCl_2_) was also used to examine the electro-oxidation reaction of hydrazine. As can be seen in Figure 5A; curve g, the Co@NCNTs-2-catalyst-modified GCE also showed no obvious catalytic activity in the absence of hydrazine, like the Co@NCNTs-1 catalyst sample. However, the Co@NCNTs-2 catalyst displayed a lower electro-oxidation response toward hydrazine (Figure 5A; curve h) due to the irregular formation or aggregated growth of Co nanoparticles on the surface walls of the NCNTs. This can vitally reflect the overall electro-oxidation response of hydrazine. In addition, Co@NCNTs-2 showed a more positive onset oxidation potential (−0.52 V; green arrow mark in Figure 5B) when compared with the Co@NCNTs-1 sample, which clearly reveals that it is ineffective towards the electro-oxidation of hydrazine. The obtained lower onset oxidation potential of the Co@NCNTs-1 catalyst electrode is indeed comparable with the recently published carbon-doped or metal/carbon-catalyst-based nanomaterials (Table S2). We have measured and fitted the anodic gas generation rate vs. the current density, which were fairly consistent with the values calculated by the four-electron hydrazine oxidation reaction at the Co@NCNTs-1 catalyst electrode (Figure S2), which suggests the formation of N_2_ during the electro-oxidation of hydrazine. Furthermore, we estimated the mass activity, i.e., the Co@NCNTs-1 electrode showed the highest mass activity (198.5 mA mg^−1^) when compared with the Co@NCNTs-2 (146.4 mA mg^−1^), and NCNTs (102.2 mA mg^−1^) electrodes. In addition, the ESCA = C_dl_/C_s_, where C_dl_ is the electrochemical double layer capacitance, which is calculated from the LSV obtained at different scan rates and C_s_ = 0.040 cm^−2^ (the specific capacitance in an alkaline electrolyte). We have calculated ECSA from C_dl_, which is uniquely associated with the active surface area of the catalyst-modified electrode; thus, the difference in the current I_D_: (I_total_-I_kinetic_) was obtained from the LSV polarization curves that were recorded using various scan rates. The C_dl_ value was found to be 0.64 mF cm^−2^, 1.73 mF cm^−2^, and 1.23 mF cm^−2^ for NCNT-, Co@NCNTs-1-, and Co@NCNTs-2-catalyst-modified electrodes, respectively. The ECSA value of the above catalyst-modified electrodes can be calculated as 16.0 cm^2^ (NCNTs), 43.25 cm^2^ (Co@NCNTs-1), and 30.75 cm^2^ (Co@NCNTs-2), which indicates that the Co@NCNTs-1-catalyst-modified electrode has a larger ECSA than the other modified electrodes, which plays a key role in its higher catalytic activity towards the enhanced electro-oxidation of hydrazine. Next, we examined the influence of NaOH concentration on the electro-oxidation of hydrazine. As displayed in Figure 5C, the LSV polarization curves were recorded at the Co@NCNTs-1-catalyst-modified electrode using different concentrations of NaOH (1.0 M, 2.0 M, 3.0, 4.0, and 5.0 M) and 0.1 M hydrazine. The electro-oxidation of hydrazine was noticeably enhanced with respect to the increasing concentrations of the NaOH solution (Figure 5C; curves a–e); however, the hydrazine onset oxidation potential slightly shifted to the positive side due to the high density of available OH^−^ ions. In other words, the efficient diffusion of hydrazine molecules was hindered due to a large amount of OH^−^ ions, signifying that the oxidation of hydrazine is purely subject to the optimized concentration of hydroxide ions (OH^−^); since an optimal concentration of OH^−^ ions (1.0 M NaOH) supports the catalytic oxidation reaction that converts hydrazine into nitrogen and water in 1.0 M NaOH (Scheme 1), this inference is in agreement with earlier studies [9,10]. Using LSV polarization curves, the Tafel plots (Figure S3) were obtained for NCNTs (Figure 5D; curve a), Co@NCNTs-2 (Figure 5D; curve b), and Co@NCNTs-1 (Figure 5D; curve c) GCEs in 1.0 M NaOH and 0.1 M Hydrazine solution. The mass transfer-associated (kinetic) current density (i_k_) was acquired from the analyzed i(_total_) value by the standard of 1/i(_total_) = 1/i_k_ + 1/i_d_. Among the catalysts, the Co@NCNTs-1 electrode (Figure 5E; curve c) displayed lowest Tafel slope value (73.1 mV dec^−1^), which is mainly based on overall electrode kinetic performances. The other catalyst electrodes, i.e., NCNTs (Figure 5D; curve a), and Co@NCNTs-2 (Figure 5D; curve b) electrodes showed higher Tafel slopes i.e., 96.0 mV dec^−1^ and 116.7 mV dec^−1^, respectively, which are generally related to mixed electrochemical reactions, such as electrode kinetics connected with mass-transfer and/or ionic conductance effects on the surface layers of catalysts. Furthermore, the kinetic current density “i” was obtained from ~−0.55 V for Co@NCNTs-1 which is larger when compared with NCNT- and Co@NCNTs-2-catalyst-modified electrodes. These results show that the Co@NCNTs-1 catalyst condensed the effect of the electrode potential (onset point) upon the activation energy of the hydrazine electro-oxidation reaction, making it considerably faster. Thus, the Co@NCNTs-1 catalyst possibly enlarged the electrode kinetics reaction in this work. In order to assess the electrode-kinetics of the hydrazine oxidation reaction on bare GCE, NCNTs, Co@NCNTs-2, and Co@NCNTs-1 electrodes, EIS analysis was conducted in 1.0 M NaOH containing 0.10 M hydrazine in the region of 1 Hz to 1 MHz and at an amplitude of 5 mV. As shown in Figure 5E, Nyquist plots with a semicircle at higher-frequency areas in all of the EIS spectra represent the existence of charge transfer resistance (R_ct_). While applying 0.1 V vs. Ag/AgCl, a semicircular portion was gradually reduced from bare GCE to catalyst-sample-modified electrodes, i.e., 53.6 Ω for bare GCE (curve a), 31.2 Ω for NCNTs (curve b), 17.7 Ω for Co@NCNTs-2 (curve c), and 8.4 Ω for Co@NCNTs-1 (curve d), demonstrating that a faster interfacial electron transfer reaction takes place during the hydrazine electro-oxidation at Co@NCNTs-1 due to its higher electronic conductivity, its large active surface area, the presence of several micro- and mesopores, and the inherent synergistic activity between N-doped CNTs vs. Co nanoparticles [46]. The acquired above EIS data was closely associated with the XPS, BET, and LSV results.

### 3.3. Amperometric Study of Hydrazine at NCNTs and Co@NCNTs

**Figure 6:**
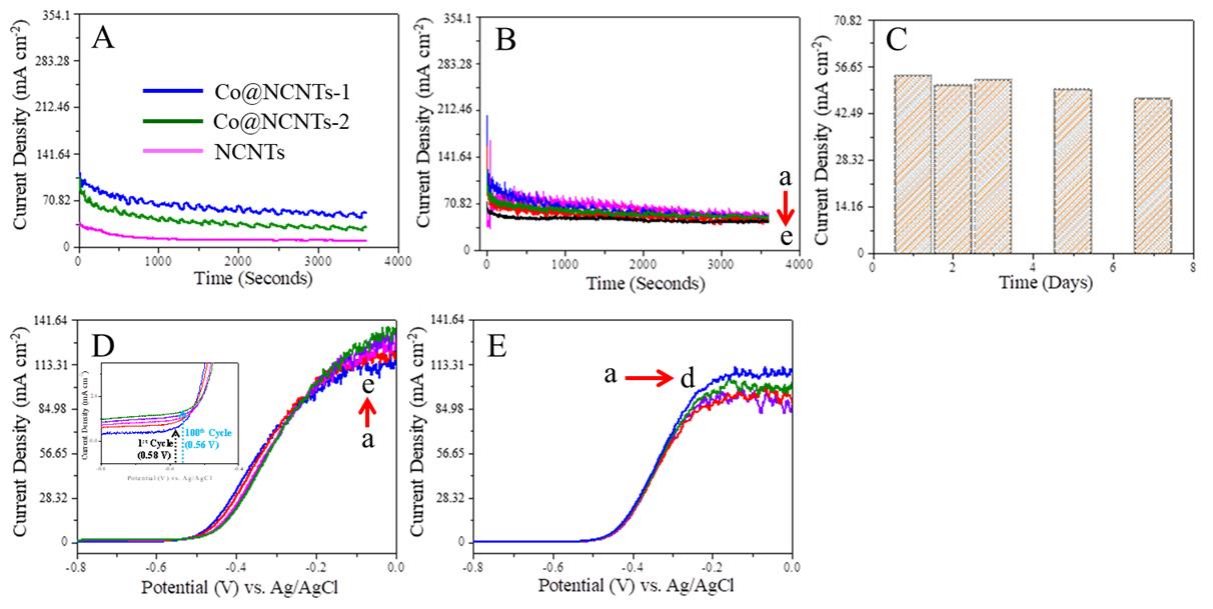
(**A**) Chronoamperomeric i-t curves obtained for NCNTs (pink line), Co@NCNTs−1 (blue line), and Co@NCNTs−2 (green line) modified GCEs in 1.0 M NaOH solution containing 0.1 M hydrazine at an applied potential of 0 V vs. Ag/AgCl under constant stirring experimental solution. (**B**) Chronoamperomeric i–t curves obtained for Co@NCNTs−1 modified GCEs at an applied potential of 0 V vs. Ag/AgCl. The i–t curves recorded after 1 day (a), 2nd day (b), 3rd day (c), 5th day (d), and 7th day (f), and corresponding bar plot showing their exact current responses (**C**). (**D**) LSV polarization curves obtained for different cycles using Co@NCNTs−1 electrode in 1.0 M NaOH solution containing 0.1 M hydrazine at a scan rate of 10 mV s^−1^ (a) 1st cycle, (b) 25th cycle, (c) 50th cycle, (d) 75th cycle, and (e) 100th cycle. (**E**) LSV polarization curves obtained for four-different Co@NCNTs−1 electrodes in 1.0 M NaOH solution containing 0.1 M hydrazine at a scan rate of 10 mV s^−1^; (blue line) 1st electrode, (green line) 2nd electrode, (red line) 3rd electrode, and (violet line) 4th electrode.

Besides the significant electro-catalytic activity, the stability and durability of the catalyst were considered as vital features for their use in DHFCs. Henceforth, the longer-term stability of the as-prepared catalysts was examined by conducting amperometric measurements in 1.0 M NaOH and 0.1 M hydrazine at an applied potential of 0 V under constant stirring for a period of 1000 s (Figure 6A). Similar to the CV results shown in Figure 5A, the Co@NCNTs-1 catalyst electrode (blue line) displayed a higher amperometric catalytic current (~4 mA at 3600 s) than the Co@NCNTs-2 (green line) and NCNT (pink line) catalyst samples. Such an enhanced catalyst-response current can be ascribed to the enhancing of active sites by Co nanoparticles and the favorable synergy effect between the catalyst materials. In addition, the Co@NCNTs-2 (green line) and NCNT (pink line) catalyst samples displayed identical responses, but the current response was comparably lower, and it was kept stable with slight variations. The steady current that was obtained demonstrated the potential durability of the Co@NCNTs-1 in using the anode material for DHFCs. In order to study the longer-storage activity of the Co@NCNTs-1 electrode, we studied the amperometric *i-t* curve analysis of Co@NCNTs-1 electrodes that were stored for one, two, three, five and seven days by testing in a solution containing 1.0 M NaOH and 0.1 M hydrazine under constant stirring (Figure 6B; curves a–e). The CA responses of the Co@NCNTs-1 electrode towards the electro-oxidation of hydrazine was predominantly stable for a week, indicated by the potential catalytic activity and corresponding bar plot (Figure 6C), which shows that only 9.2% of activity was lost after a week from its fresh preparation, demonstrating noticeable long-storage stability. The electro-oxidation of hydrazine was continuously tested at Co@NCNTs-1 in 1.0 M NaOH and 0.1 M hydrazine using the LSV curve stability. LSV curves showed only a 3.7% loss during the oxidation process at 100 cycles (Figure 6D curves a–e), demonstrating the high-stability (i.e., no Co-nanoparticle agglomeration) and considerable durability of the as-synthesized low-cost carbon-based-catalyst samples. We carried out CV scans after the extended chronoamperometry analysis, and the corresponding SEM and TEM characterizations were shown in Supporting Information Figure S4. After the extended chronoamperometry analysis, the CV curve retained its original structure, despite a slight positive shift in the onset oxidation potential (−0.48 V) and a small decrease in its oxidation current response (7.4 mA). The Co loading was calculated after the extended chronoamperometry analysis and it was about 6.57%. Finally, we prepared four different Co@NCNTs-1 electrodes under identical conditions, and carried out reproducibility measurements (Figure 6E). The successive LSV polarization responses of all four Co@NCNTs-1 electrodes showed narrowly identical results toward the electro-oxidation of hydrazine, displaying distinctive repeating LSV polarization curves. The overall experimental results showed that the Co@NCNTs-1 electrode could be used as an alternative non-noble metal-based catalyst material for the DHFC anode.

## 4. Conclusions

In the present study, Co nanoparticles were integrated on the tubular wall surface of an N-doped carbon network with a higher active surface area and mesoporosity. Owing to the abundance of active sites accessible on its surface and the synergistic effect between Co nanoparticles and N-doped carbon tube-like network, the newly obtained Co@NCNTs-1 catalyst material revealed a higher catalytic activity towards electro-oxidation of hydrazine in an alkaline medium. In particular, the Co@NCNTs-1 catalyst electrode showed substantial activity and remarkable stability toward the electro-oxidation of hydrazine due to: (i) spherical Co nanoparticles that were constantly introduced on the surface of the NCNTs, and which produced several catalytic-active sites; (ii) porous N-doped carbon nanotubes with a confirmed interaction between the electrolyte and Co@NCNTs-1 due to its higher surface area and abundant micro- and mesoporous surface; (iii) the prominent synergistic effect between the Co nanoparticles and NCNTs, and the existence of higher pyridinic-N and pyrrolic-N (52.4%) functionalities, which significantly increase the electro-catalytic activity of hydrazine. The obtained Co@NCNTs-1 exhibited a superior electro-catalytic property, which can be employed as a potential anode material for DHFC applications. In addition, cobalt-nanoparticle-integrated carbon nanomaterials are considered as efficient candidates in the removal of organic pollutants, especially with regard to the activation of peroxymonosulfate (PMS) or sulfite to degrade organic dye pollutants (rhodamine B, methyl orange, etc.) in water.

## Supplementary Materials

The following are available online at https://www.mdpi.com/article/10.3390/nano11112857/s1, Figure S1: SEM image of NCNTs sample without Co nanpoparticles, Figure S2: The variation of anodic gas generation rate as a function of current density obtained using Co@NCNTs-1 catalyst electrode, Figure S3: Tafel plots taken on a NCNTs (curve a), Co@NCNTs-2 (curve b), and Co@NCNTs-1 (curve c) catalyst samples modified GCE electrodes resulting from their respective LSV curves, Figure S4: CV curve obtained for the extended chronoamperometry analysis using Co@NCNTs-1 catalyst electrode in 1.0 M NaOH solution containing 0.1 M hydrazine at a scan rate of 10 mV s^-1^, and corresponding SEM (B), and TEM images (C) displaying the stability characterization using Co@NCNTs-1 catalyst electrode, Table S1: Comparison of BET surface area of Co@NCNTs-1 with the results reported in the literature, Table S2: Comparison of lower-onset oxidation potential obtained at Co@NCNTs-1 catalyst electrode with recently published metal/carbon catalysts based nanomaterials.
