# Supplementary material for: Co Nanoparticle-Encapsulated Nitrogen-Doped Carbon Nanotubes as an Efficient and Robust Catalyst for Electro-Oxidation of Hydrazine
Source: Nanomaterials (Basel). 2021 Oct 26;11(11):2857. doi: 10.3390/nano11112857 (PMC8619281; doi:10.3390/nano11112857)
Supplement: Supplementary file 1 [file nanomaterials-11-02857-s001.zip › nanomaterials-1418613-supplementary.pdf]

nanomaterials

Supplementary Materials

Hui Wang [1,2], Qing Dong [2], Lu Lei [1,*], Shan Ji [1], Palanisamy Kannan [1,*], Palaniappan Subramanian [3,*] and Amar Prasad Yadav [4]

[1] College of Biological, Chemical Sciences and Engineering, Jiaxing University, Jiaxing 314001, China; wangh@qust.edu.cn (H.W.); jishan@mail.zjxu.edu.cn (S.J.)
[2] College of Chemical Engineering, Qingdao University of Science and Technology, Qingdao 266042, China; dongqing125800@163.com
[3] New Technologies–Research Center, University of West Bohemia, Plzeň 30100, Czech Republic;
[4] Central Department of Chemistry, Tribhuvan University, Kirtipur-44613, Kathmandu, Nepal; amar2y@yahoo.com
* Correspondence: Authors: lulei@zjxu.edu.cn (L.L.); ktpkannan@zjxu.edu.cn (P.K.); palans@ntc.zcu.cz (P.S.); Tel.: +86-19884388523 (L.L.); +86-19857386580 (P.K.); Fax: +86-573-83643264 (L.L.)

Citation: Wang, H.; Dong, Q.; Lei, L.; Ji, S.; Kannan, P.; Subramanian, P. Yadav, A.P. Co Nanoparticles Encapsulated Nitrogen-Doped Carbon Nanotubes as an Efficient and Robust Catalyst for Electro-Oxidation of Hydrazine. *Nanomaterials* **2021**, *11*, 2857. https://doi.org/10.3390/nano11112857

Academic Editor: Sónia Carabineiro

Received: 27 September 2021
Accepted: 23 October 2021
Published: 26 October 2021

Publisher's Note: MDPI stays neutral with regard to jurisdictional claims in published maps and institutional affiliations.

Copyright: © 2021 by the authors. Licensee MDPI, Basel, Switzerland. This article is an open access article distributed under the terms and conditions of the Creative Commons Attribution (CC BY) license (http://creativecommons.org/licenses/by/4.0/).

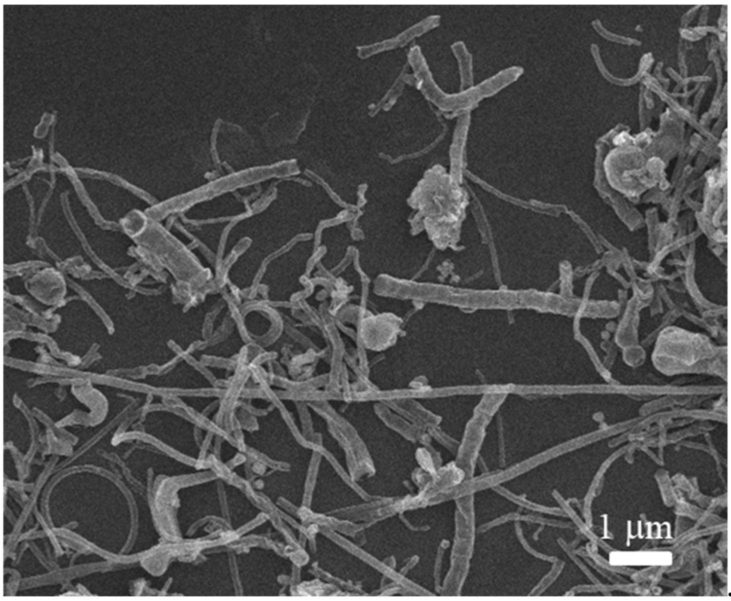

Figure S1. SEM image of NCNTs sample without Co nanpoparticles.

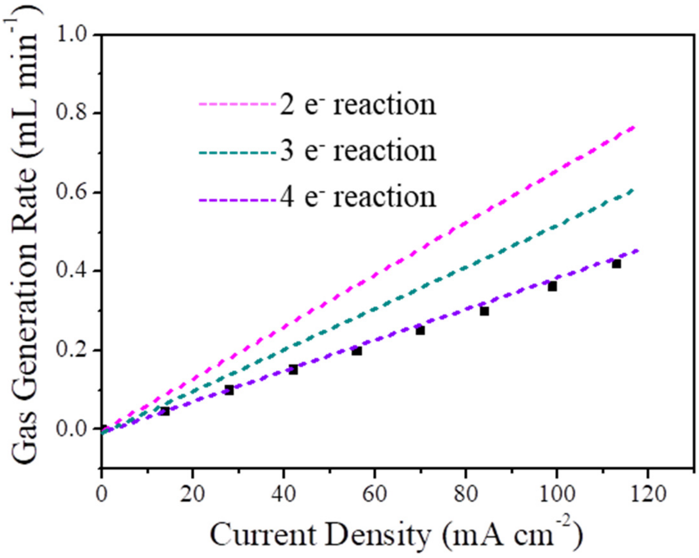

Figure S2. The variation of anodic gas generation rate as a function of current density obtained using Co@NCNTs-1 catalyst electrode.

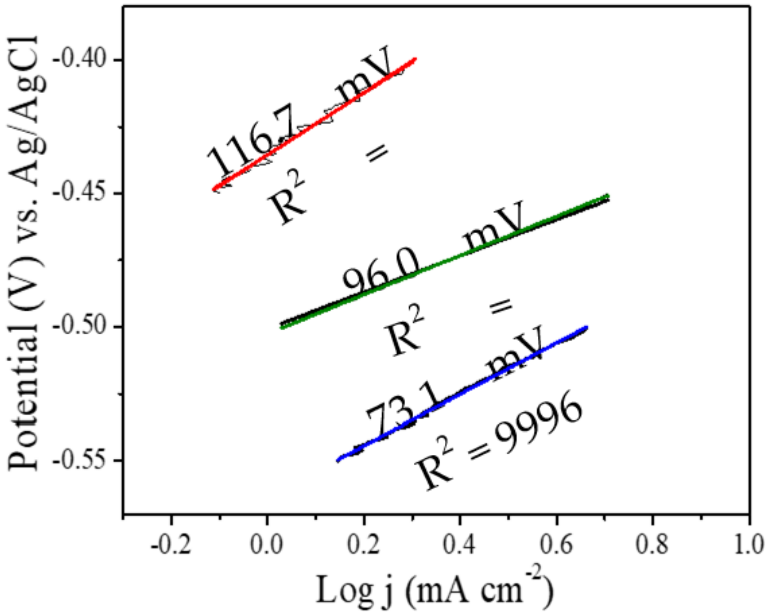

Figure S3. Tafel plots taken on a NCNTs (curve a), Co@NCNTs-2 (curve b), and Co@NCNTs-1 (curve c) catalyst samples modified GCE electrodes resulting from their respective LSV curves.

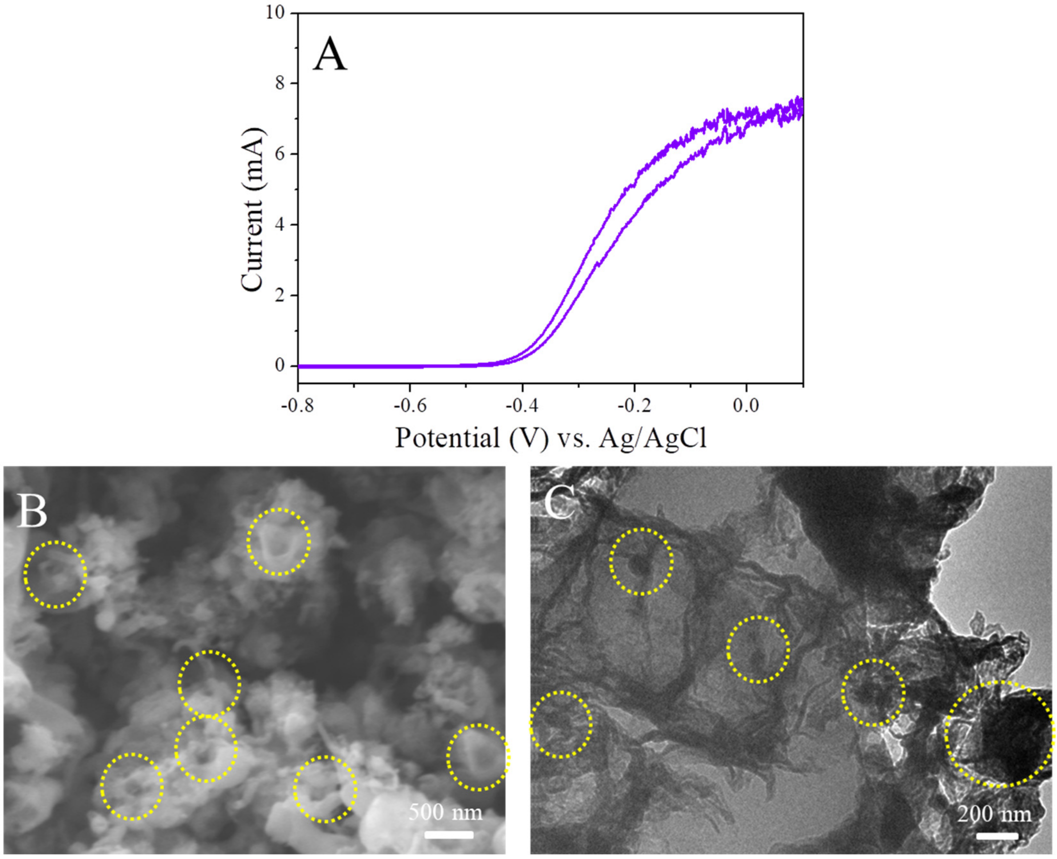

Figure S4. CV curve obtained for the extended chronoamperometry analysis using Co@NCNTs-1 catalyst electrode in 1.0 M NaOH solution containing 0.1 M hydrazine at a scan rate of 10 mV s^{-1}, and corresponding SEM (B), and TEM images (C) displaying the stability characterization using Co@NCNTs-1 catalyst electrode.

Table S1. Comparison of BET surface area of Co@NCNTs-1 with the results reported in the literature.

**Scheme.**	**Name of the Nanomaterials**	**BET surface area/m^2g^{-1}**	**References**
1	Co@NCNTs-1	247.2	This Work
2	Fe/Fe_3C@NGL-NCNT	210	[1]
3	PL-bio-C	201.3	[2]
4	N-Fe/N/C-CNT	173.9	[3]
5	$ZnCo_2O_4$/N-CNT	159	[4]
6	MWCNT@S–N–C	121	[5]
7	Fe@C-NG/NCNTs	117.6	[6]

Table S2. Comparison of lower-onset oxidation potential obtained at Co@NCNTs-1 catalyst electrode with recently published metal/carbon catalysts based nanomaterials.

**Scheme.**	**Name of the Nanomaterials**	**Onset Oxidation Potential (V)**	**References**
1	NiO-NWs@N-HCNSs/GCE	+0.05	[7]
2	P-$CuNi_2$/C	-0.55	[8]
3	h-$MnFe_2O_4$ NPs/N-rGO	-0.20	[9]
4	Fe@Cu-P/C	-0.15	[10]
5	PR/$NiFe_2O_4$	+0.50	[11]
6	Co@NCNTs	-0.58	This Work

References

1. Li, J.-S.; Li, S.-L.; Tang, Y.-J.; Han, M.; Dai, Z.-H.; Bao, J.-C.; Lan, Y.-Q. Nitrogen-doped Fe/Fe3C@graphitic layer/carbon nanotube hybrids derived from MOFs: efficient bifunctional electrocatalysts for ORR and OER. *Chem. Commun.* **2015**, *51*, 2710–2713.
2. Wu, Y.; Wang, Y.; Xiao, Z.; Li, M.; Ding, Y.; Qi, M.-l., Electrocatalytic oxygen reduction by a Co/Co3O4@N-doped carbon composite material derived from the pyrolysis of ZIF-67/poplar flowers. *RSC Adv.* **2021,** *11*, 2693–2700.
3. Chen, P.; Zhou, T.; Xing, L.; Xu, K.; Tong, Y.; Xie, H.; Zhang, L.; Yan, W.; Chu, W.; Wu, C.; Xie, Y. Atomically Dispersed Iron–Nitrogen Species as Electrocatalysts for Bifunctional Oxygen Evolution and Reduction Reactions. *Angew. Chem. Int. Ed.* **2017,** *56*, 610–614.
4. Liu, Z.-Q.; Cheng, H.; Li, N.; Ma, T. Y.; Su, Y.-Z. ZnCo2O4 Quantum Dots Anchored on Nitrogen-Doped Carbon Nanotubes as Reversible Oxygen Reduction/Evolution Electrocatalysts. *Adv. Mater.* **2016**, *28*, 3777–3784.
5. Zhu, Q.; Lin, L.; Jiang, Y.-F.; Xie, X.; Yuan, C.-Z.; Xu, A.-W. Carbon nanotube/S–N–C nanohybrids as high performance bifunctional electrocatalysts for both oxygen reduction and evolution reactions. *New J. Chem.* **2015**, *39*, 6289–6296.
6. Wang, Q.; Lei, Y.; Chen, Z.; Wu, N.; Wang, Y.; Wang, B.; Wang, Y. Fe/Fe3C@C nanoparticles encapsulated in N-doped graphene–CNTs framework as an efficient bifunctional oxygen electrocatalyst for robust rechargeable Zn–air batteries. *J. Mater. Chem. A* **2018**, *6*, 516–526.
7. Taghaddosi, S.; Rezaee, S.; Shahrokhian, S. Facile synthesis of N-doped hollow carbon nanospheres wrapped with transition metal oxides nanostructures as non-precious catalysts for the electro-oxidation of hydrazine. *J. Electroanal. Chem.* **2020,** *873*, 114437.
8. Wang, W.; Wang, Y.; Liu, S.; Yahia, M.; Dong, Y.; Lei, Z. Carbon-supported phosphatized CuNi nanoparticle catalysts for hydrazine electrooxidation. *Int. J. Hydrog. Energy* **2019**, *44*, 10637–10645.

9. Khilari, S.; Pradhan, D. MnFe2O4@nitrogen-doped reduced graphene oxide nanohybrid: an efficient bifunctional electrocatalyst for anodic hydrazine oxidation and cathodic oxygen reduction. *Catal. Sci. Technol.* **2017**, *7*, 5920–5931.
10. Wang, Y.; Liu, X.; Tan, T.; Ren, Z.; Lei, Z.; Wang, W. A phosphatized pseudo-core-shell Fe@Cu-P/C electrocatalyst for efficient hydrazine oxidation reaction. *J. Alloys Compd.* **2019**, *787*, 104–111.
11. Lashkenari, M. S.; Shahrokhi, B.; Ghorbani, M.; falah, J.; Rostami, H. Polyrhodanine/NiFe2 O4 nanocomposite: A novel electrocatalyst for hydrazine oxidation reaction. *Int. J. Hydrog. Energy* **2018**, *43*, 11244–11252.
